# Glymphatic Pathway Dysfunction in Mild Cognitive Impairment: A Systematic Review and Meta‐Analysis Using Diffusion Tensor Imaging Along the Perivascular Space

**DOI:** 10.1002/cns.70695

**Published:** 2025-12-19

**Authors:** Sadegh Ghaderi, Sana Mohammadi, Ali Fathi Jouzdani, Amir Mahmoud Ahmadzadeh

**Affiliations:** ^1^ Neuromuscular Research Center, Department of Neurology Shariati Hospital, Tehran University of Medical Sciences Tehran Iran; ^2^ Department of Neuroscience and Addiction Studies, School of Advanced Technologies in Medicine Tehran University of Medical Sciences Tehran Iran; ^3^ Department of Medical Sciences, School of Medicine Iran University of Medical Sciences Tehran Iran; ^4^ School of Medicine Hamadan University of Medical Sciences Hamadan Iran; ^5^ School of Cognitive Sciences Institute for Research in Fundamental Sciences (IPM) Tehran Iran; ^6^ Neuroscience & Neoplasia Artificial Intelligence Research Group (NAIRG), Department of Neuroscience, School of Science and Advanced Technologies in Medicine Hamadan University of Medical Sciences Hamadan Iran; ^7^ Department of Radiology, School of Medicine Mashhad University of Medical Sciences Mashhad Iran

**Keywords:** diffusion tensor imaging, glymphatic system, mild cognitive impairment, perivascular space

## Abstract

**Objective:**

To evaluate glymphatic dysfunction in mild cognitive impairment (MCI) using the diffusion tensor imaging along the perivascular space (DTI‐ALPS) index.

**Methods:**

Using PRISMA 2020, we searched PubMed, Web of Science, Scopus, and Embase (until May 10, 2025) for studies comparing the ALPS index in MCI patients versus healthy controls.

**Results:**

The pooled meta‐analysis of 18 studies (1133 MCI patients, mean age 69.3 years; 1275 HCs) revealed a significantly lower ALPS index in MCI patients than in HCs (Cohen's *d* = −0.70; 95% CI: −1.10, −0.29; *p* < 0.001), with high heterogeneity (*I* ^2^ = 95.3%). Sensitivity analyses confirmed the robustness of the effect and a cumulative meta‐analysis showed a consistent negative trend from 2021 to 2025. Subgroup analyses showed trends toward larger effect sizes in older patients (> 68 years, *d* = −0.83) and in studies using ≥ 64 diffusion directions (*d* = −0.99), although formal tests for subgroup differences were not statistically significant. Small‐study effects were found (Egger's *p* = 0.004; Begg's *p* = 0.019), but the studies had high methodological quality. Trim‐and‐fill analysis added six studies, adjusting pooled Cohen's *d* from −0.70 to −0.98, indicating potential overestimation from publication bias.

**Conclusions:**

These findings support the utility of the ALPS index as a noninvasive biomarker for early glymphatic dysfunction in cognitive decline.

## Graphical abstract

Our meta‐analysis of 18 studies identifies a significant reduction in the DTI‐ALPS index in mild cognitive impairment (Cohen's *d* = −0.70), highlighting glymphatic dysfunction as a biomarker for early neurodegeneration.

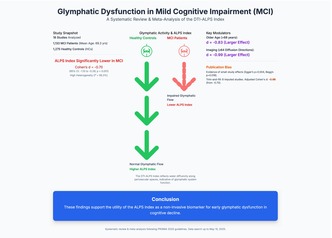

## 1 Introduction

The glymphatic system operates as a perivascular space (PVS) that is crucial for clearing metabolic waste from the central nervous system (CNS) [1, 2]. Its function mirrors that of the glymphatic pathway found in peripheral tissues. It is crucial to clear waste materials and proteins and prevent their accumulation [3]. The clearance process is particularly important in neurodegenerative conditions [4].

Mild cognitive impairment (MCI) serves as an essential intermediate stage between normal cognitive function and dementia, and poses a significant risk factor for the development of dementia [5]. While some patients with MCI advance to dementia, others maintain cognitive stability or even revert to normal functioning [6]. Gaining deeper insights into these risks can also enhance our understanding of the underlying mechanisms of cognitive decline in MCI, ultimately contributing to more effective and personalized treatment approaches for neurodegenerative disorders [7]. MCI is also associated with an increased volume fraction of PVSs; enlarged PVSs may disrupt cerebrospinal fluid (CSF)/interstitial fluid (ISF) flow and impair waste clearance [8].

Gadolinium‐based contrast agents or radioactive tracers administered intrathecally or intravenously are the most accurate methods for assessing glymphatic system functionality in living individuals [9]. Despite their precision, these techniques are invasive, making them impractical for large‐scale human studies. Therefore, researchers are focusing on developing less invasive and more accessible biomarkers to effectively evaluate glymphatic system function.

Advanced diffusion MRI assesses glymphatic function and cerebral fluid dynamics [10, 11, 12], with each targeting distinct compartments. Low *b*‐value imaging (*b* = 100–200 s/mm^2^) reveals anisotropic CSF motion near arteries and impaired CSF flow in normal‐pressure hydrocephalus [13, 14, 15, 16, 17]. Dynamic diffusion‐weighted imaging (DWI) with cardiac gating captures pulsatile CSF motion in the PVSs, demonstrating systole‐driven flow in rodents and humans [18, 19]. Intravoxel incoherent motion (IVIM) quantifies incoherent CSF flow in the ventricles and subarachnoid spaces, showing reduced flow in NPH [20, 21], while spectral diffusion analysis isolates intermediate components representing interstitial/PVS fluid [22]. Free‐water (FW) correction mitigates biases in DTI metrics by accounting for extracellular fluid changes during aging and neurodegeneration [23, 24, 25]. Higher‐order models, such as diffusion kurtosis imaging (DKI) and neurite orientation dispersion and density imaging (NODDI), detect interstitial volume changes and free‐water alterations in idiopathic NPH and small‐vessel disease [26, 27]. Complementary methods include abnormal white matter signal volume as a glymphatic marker [28], enlarged PVS (ePVS) quantification linked to vascular pathology [29, 30, 31, 32, 33], and advanced sequences, such as T2‐weighted 3D fast spin echo and dynamic DWI for CSF mobility [19, 34]. Collectively, these techniques provide multifaceted insights into glymphatic dynamics, although standardization and validation remain critical for clinical translation.

Beyond the diffusion MRI techniques [10], diffusion‐based magnetic resonance imaging (MRI) is a novel imaging technique for evaluating the brain microstructure by analyzing the movement of water molecules at the microscopic level. Among these techniques, diffusion tensor imaging (DTI) is widely utilized [35]. A specific adaptation known as DTI along the PVS (DTI‐ALPS) was introduced to measure radial diffusion in the PVS of the deep medullary veins [36, 37, 38]. This provided an indirect measure of the ALPS index [36]. The reliability of the ALPS index as a noninvasive metric for glymphatic pathway function has been validated through comparisons with intrathecal gadolinium‐based contrast agent tracer studies [36, 38].

Therefore, our review aimed to conduct a comprehensive systematic review and meta‐analysis on glymphatic pathway dysfunction in patients with MCI compared with healthy controls (HCs) using DTI‐ALPS.

## 2 Methods

### 2.1 Search, Eligibility Criteria, and Screening

We adhered to the Preferred Reporting Items for Systematic Reviews and Meta‐Analyses (PRISMA) 2020 guidelines [39]. Four major databases, namely PubMed, Web of Science, Scopus, and Embase, were systematically searched on May 10, 2025. The search strategy was adapted according to the PECO (Population: patients with MCI, Exposure: DTI‐ALPS, Comparison: HCs, Outcome: ALPS index). Keywords related to the “glymphatic system,” “DTI‐ALPS,” and “MCI” were used along with the appropriate Boolean operators. Table S1 details the search strategies employed across the four databases to identify studies that investigated the glymphatic system, diffusion tensor imaging (DTI or DTI‐ALPS), and MCI.

We included original papers that compared the ALPS index between patients with MCI, regardless of type, and HCs. To ensure high‐quality evidence, we only included peer‐reviewed studies. Irrelevant articles, conference abstracts/papers, reviews, animal studies, letters/editorials, and preprint studies were excluded.

We imported the initially identified studies into the Rayyan platform (https://www.rayyan.ai/) and EndNote software (v.21), and performed deduplication. Next, the titles and abstracts of the remaining studies were independently screened by three reviewers (S.G., A.M.A., and A.F.J.). Relevant articles retrieved from this step were reviewed by two experienced authors (S.G. and S.M.). Discrepancies were resolved through discussion.

### 2.2 Data Extraction

Data extraction was independently performed by two reviewers (A.F.J. and A.M.A.). Key study characteristics, including first author name, publication year, country, DTI information, demographic data of the subjects, cognitive scores of patients with MCI, and the ALPS index, were extracted from the included studies. For studies that did not report ALPS indices in the text, we used PlotDigitizer (https://plotdigitizer.com/app) to extract them from figures when available. Discrepancies were addressed through joint discussions.

### 2.3 Publication Bias Assessment

We visualized funnel plots to identify any asymmetry that might suggest the selective reporting of studies. Publication bias was assessed quantitatively using Egger's regression [40] and Begg's test [41]. Statistical significance was set at *p* < 0.05. The trim‐and‐fill method was subsequently employed to assess and adjust for potential biases.

### 2.4 Risk of Bias Assessment

For quality assessment, we used the modified Newcastle‐Ottawa Scale (NOS) based on the Ottawa checklist for cross‐sectional studies [42, 43], which evaluates the quality of studies based on selection, comparability, and outcome criteria. Studies with a score of 9 were very good quality, 7–8 were good quality, 5–6 were satisfactory quality, and 0–4 were unsatisfactory quality [44]. This step was conducted by two reviewers independently (A.M.A. and A.F.J.). The initial scores were further reviewed by two experienced authors (S.G. and S.M.), and the final scores were determined through joint discussion.

### 2.5 Statistical Analysis

We applied a random‐effects model to compare the ALPS index between patients with MCI and HCs. Thresholds of 0.2, 0.5, and 0.8 were utilized to interpret the magnitude of the differences [45]. When original data needed conversion suitable for our analysis, such as combining data from two separate databases [46], we used the formula outlined in the Cochrane Handbook of Systematic Reviews of Interventions, with calculations performed via the website (https://meta‐converter.com/) [47]. The *I* ^2^ statistic was used to quantify between‐study heterogeneity, categorizing it as low (0%–25%), moderate (25%–50%), high (50%–75%), or very high (75%–100%) [48, 49]. To investigate the potential sources of heterogeneity, we performed various subgroup analyses and evaluated the effects of several factors on ALPS, except for echo time, due to inconsistencies in reporting and insufficient data. These factors included hemisphere, sex, age, coil, male ratio, number of directions, and Mini‐Mental State Examination (MMSE) score. Sensitivity analysis was conducted using the leave‐one‐out method to assess the impact of each study on the pooled estimate and to evaluate the robustness of the overall findings. Data analysis was performed using the STATA software version 17 (StataCorp, College Station, TX, USA).

## 3 Results

### 3.1 Overview of Results

**FIGURE 1:**
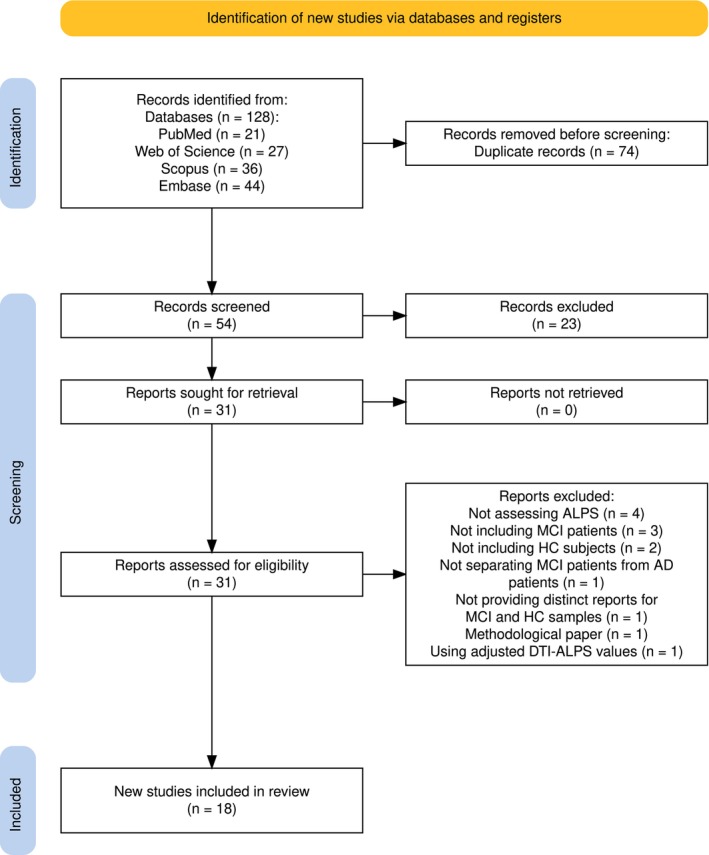
PRISMA flow diagram illustrating the systematic study selection process.

The PRISMA 2020 flowchart outlines the systematic study selection process for the review (Figure 1) [39]. Initially, 128 records were identified in PubMed (*n* = 21), Web of Science (*n* = 27), Scopus (*n* = 36), and Embase (*n* = 44). After 74 duplicate records were removed, 54 studies were screened. Of these, 23 were excluded on the basis of their title/abstract relevance, leaving 31 reports for full‐text retrieval. All 31 reports were successfully retrieved and assessed for their eligibility. Eight reports were excluded for the following reasons: not assessing the ALPS index (*n* = 4), lack of MCI patients (*n* = 3), no inclusion of HCs (*n* = 2), failure to distinguish MCI from Alzheimer's disease (AD) (*n* = 1), not providing distinct data for the MCI and HC groups (*n* = 1), being a methodological study (*n* = 1), and using adjusted DTI‐ALPS values (*n* = 1). Ultimately, 18 new studies met the inclusion criteria and were included in the present review (Table 1).

**TABLE 1:** Summary of studies investigating glymphatic pathway dysfunction using Diffusion Tensor Imaging Along the Perivascular Space (DTI‐ALPS) in patients with mild cognitive impairment (MCI) compared to healthy controls (HCs). The type of all MCI is multiple‐domain amnestic MCI (a‐MCI‐MD).

Author/year	Field strength (Tesla)	Coil	*b*‐value (s/mm^2^)	Directions	Healthy controls		MCI				DTI‐ALPS results	
					Subjects (male)	Age (SD)	Domain	Patients (male)	Age (SD)	MMSE (SD)	MCI DTI‐ALPS (SD)	HCs DTI‐ALPS (SD)
Hu et al. (2025) [50]	3	64	1000	64	35 (21)	64.03 (11.26)	T2‐DM	35 (23)	68.20 (9.15)	27.60 (1.85)	1.24 (0.12)	1.53 (0.05)
Guo et al. (2025) [46]	3	64	1000	41, 64	60 (29)	71.35 (7.31)	AD	114 (68)	72.05 (6.55)	27.15 (1.57)	1.25 (0.13)	1.33 (0.16)
Huang et al. (2024) [51]	3	32	1000	NR	235 (85)	68.7 (3.85)	AD	137 (83)	68.20 (5.17)	NR	1.24 (0.20)	1.28 (0.19)
Li et al. (2025) [52]	3	NR	1000	64	32 (16)	64.63 (6.56)	NR	28 (12)	67.14 (7.24)	27.25 (1.58)	1.25 (0.18)	1.35 (0.17)
Bao et al. (2025) [53]	3	NR	1000	NR	252 (112)	72.85 (6.8)	AD	266 (151)	74.01 (7.50)	NR	1.39 (0.19)	1.47 (0.17)
Sacchi et al. (2024) [54]	3	NR	1000 2000	32	23 (14)	68.5 (12.25)	MCI	17 (8)	73 (5.66)	26.67 (2.37)	1.32 (0.19)	1.35 (0.28)
Sun et al. (2024) [55]	3	32	1000	64	18 (9)	63.44 (6.92)	AD	23 (8)	67.74 (6.99)	20 (5.53)	1.23 (0.13)	1.35 (0.12)
Pang et al. (2024) [56]	3	32	1000	64	35 (16)	62.94 (5.22)	PD	35 (18)	65.77 (3.86)	23.00 (1.50)	1.45 (0.26)	1.50 (0.31)
Zhou et al. (2024) [57]	3	32	1000	120	31 (9)	61.61 (5.8)	MCI	54 (17)	64 (6.09)	24.67 (2.28)	1.4 (0.16)	1.5 (0.20)
Wang et al. (2024) [58]	3	15	800	48	26 (9)	62.73 (8.56)	PD	29 (18)	61.03 (9.88)	27.99 (1.40)	1.30 (0.15)	1.33 (0.14)
Huang et al. (2024) [51]	3	32	NR	NR	235 (85)	68.7 (3.85)	AD	137 (83)	68.2 (5.17)	NR	1.24 (0.19)	1.28 (0.18)
Hong et al. (2024) [59]	3	NR	1000	48	40 (17)	75.2 (8.4)	AD	26 (7)	75.4 (9.20)	NR	1.25 (0.16)	1.35 (0.15)
Zhang et al. (2024) [60]	3	64	1000	104	26 (11)	63.4 (7.4)	AD	15 (7)	66.0 (8.6)	19.3 (6.20)	1.48 (0.13)	1.57 (0.16)
Zhong et al. (2023) [61]	3	NR	NR	NR	111 (54)	68.6 (7.5)	AD	120 (52)	72 (7.10)	24.2 (2.90)	2.15 (0.23)	2.21 (0.21)
Liang et al. (2023) [62]	3	NR	1000	NR	28 (11)	67.75 (6.09)	AD	18 (9)	71.28 (8.66)	25.22 (2.78)	1.19 (0.07)	1.44 (0.07)
Kamagata et al. (2022) [63]	3	NR	1000	41	31 (14)	73.86 (4.91)	AD	44 (26)	73.38 (5.68)	27.47 (1.94)	1.33 (0.17)	1.34 (0.11)
Chen et al. (2021) [64]	3	NR	1000	13	47 (15)	61.53 (4.75)	PD	25 (8)	63.8 (8.70)	27.08 (1.32)	1.32 (0.29)	1.45 (0.14)
Steward et al. (2021) [65]	3	12	1000	30	10 (5)	73.8 (4.89)	AD	10 (4)	76.1 (7.90)	28.3 (1.57)	1.47 (0.20)	1.53 (0.20)

*Note:* Data include DTI‐ALPS indices (mean ± SD) for patient groups and HCs. Missing data are denoted as NR.

Abbreviations: AD, Alzheimer's disease; MMSE, Mini‐Mental State Examination; NR, not reported; PD, Parkinson's disease; SD, standard deviation; T2‐DM, type‐2 diabetes mellitus.

The pooled meta‐analysis of 18 studies, which included 1133 patients with MCI (mean age: 69.3 years; 604 male) and 1275 HCs. Table 1 provides a detailed summary of the studies that investigated the use of the DTI‐ALPS index in individuals with MCI and HCs. These studies consistently employed a magnetic field strength of 3 Tesla and predominantly used *b*‐values of 1000 s/mm^2^. DTI protocols varied in terms of diffusion directions and coil configurations. Among the studies that reported these parameters, the most frequently utilized were 64 diffusion directions and 32‐channel coils. MCI represents an intermediate stage between normal cognitive functioning and the onset of dementia [66]. MMSE scores were reported for patients with MCI, with a mean of 25.42.

### 3.2 Meta‐Analysis

#### 3.2.1 Pooled Meta‐Analysis

**FIGURE 2:**
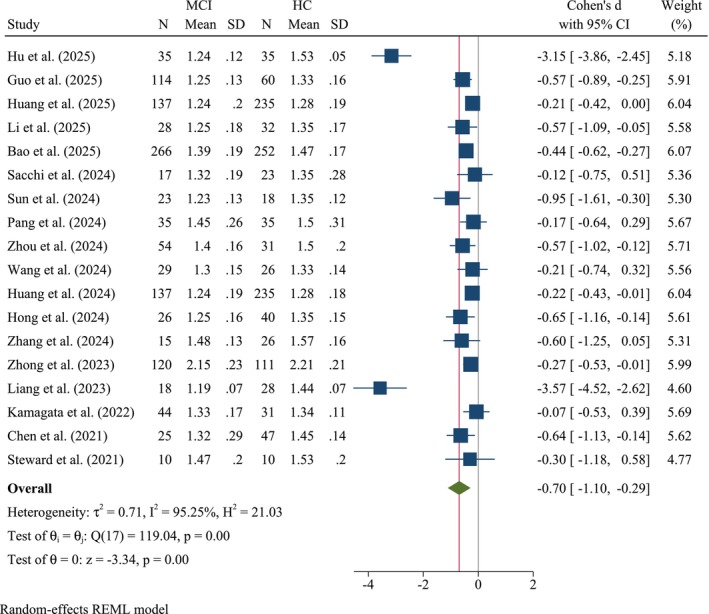
Forest plot of the meta‐analysis comparing diffusion tensor imaging along the perivascular space (DTI‐ALPS) index between patients with mild cognitive impairment (MCI) and healthy controls (HCs).

The pooled meta‐analysis results (Figure 2) revealed statistically significant moderate standardized mean differences (SMDs), indicating a lower DTI‐ALPS index in individuals with MCI than in HCs. Using a random‐effects restricted maximum likelihood model, Cohen's *d* was −0.70 (95% CI: −0.1.10, −0.29, *z* = −3.34, *p* < 0.001, *k* = 18). Heterogeneity was high, with *τ* ^2^ = 0.71, *I* ^2^ = 95.25%, and *H* ^2^ = 21.03. Cochran's *Q* test for heterogeneity was not significant (*Q*(17) = 119.04, *p* < 0.001), supporting homogeneity among SMDs.

#### 3.2.2 Cumulative Year of Publication and Sensitivity Analysis

**FIGURE 3:**
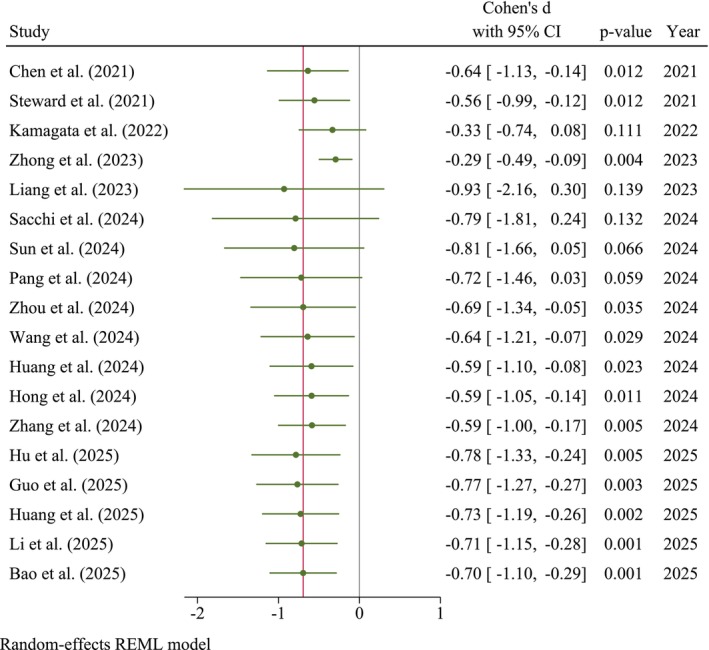
Cumulative forest plot stratified by publication year.

The analysis of the cumulative publication years (Figure 3) revealed a generally consistent and statistically significant negative effect size from 2021 to 2025. This represents an almost consistent decrease in the ALPS index in patients with MCI compared with HCs. This temporal progression suggests that with the accumulation of studies, the findings have increasingly converged toward a consistent and statistically significant negative effect.

**FIGURE 4:**
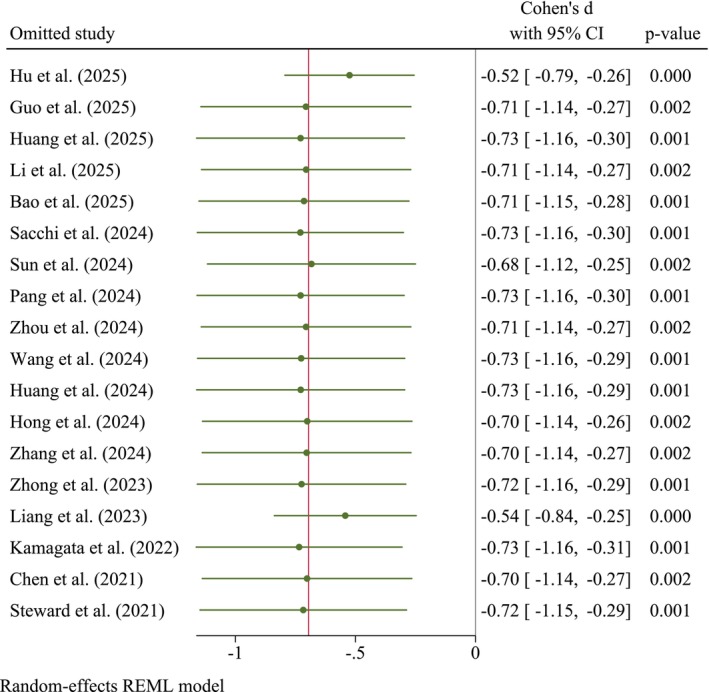
Leave‐one‐out sensitivity analysis.

Furthermore, the sensitivity analysis indicated the robustness of this finding (Figure 4). Our results indicate that omitting individual studies did not substantially alter the overall effect size or statistical significance. Notably, a leave‐one‐out sensitivity analysis (Figure 4) confirmed that the overall result was robust and not unduly driven by any single study, including those reporting particularly large effect sizes such as Hu et al. [50] and Liang et al. [62]. The narrow range of the effect sizes and consistently *p* < 0.01 analyses suggest that the primary findings are robust and not overly influenced by any single study included in the meta‐analysis.

#### 3.2.3 Age Subgroup

**FIGURE 5:**
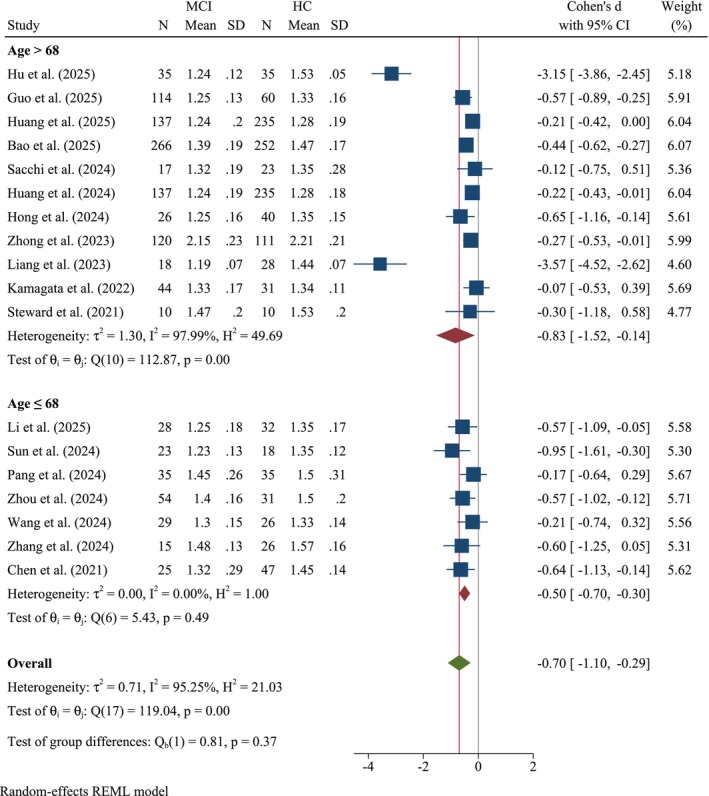
Forest plot of the meta‐analysis stratified by age (cut‐off: 68 years).

Subgroup analysis stratified by the mean age of patients with MCI (cut‐off: 68 years) revealed distinct patterns of glymphatic dysfunction (Figure 5). For patients aged > 68 years, Cohen's *d* was −0.83 (95% CI: −1.52, −0.14, *k* = 11), indicating a significant reduction in ALPS index values with high heterogeneity (*τ* ^2^ = 1.30, *I* ^2^ = 97.99%, *Q*(10) = 112.87, *p* < 0.001). In contrast, participants aged ≤ 68 years exhibited a lower SMD (Cohen's *d* = −0.50, 95% CI: −0.70, −0.30, *k* = 7), suggesting a moderate decline in glymphatic activity with low heterogeneity, which was not significant (*τ* ^2^ = 0.00, *I* ^2^ = 0.00%, *Q*(6) = 5.43, *p* = 0.49). Subgroup differences were not statistically significant (*Q*_b_(1) = 0.81, *p* = 0.37).

#### 3.2.4 Coil Subgroup

**FIGURE 6:**
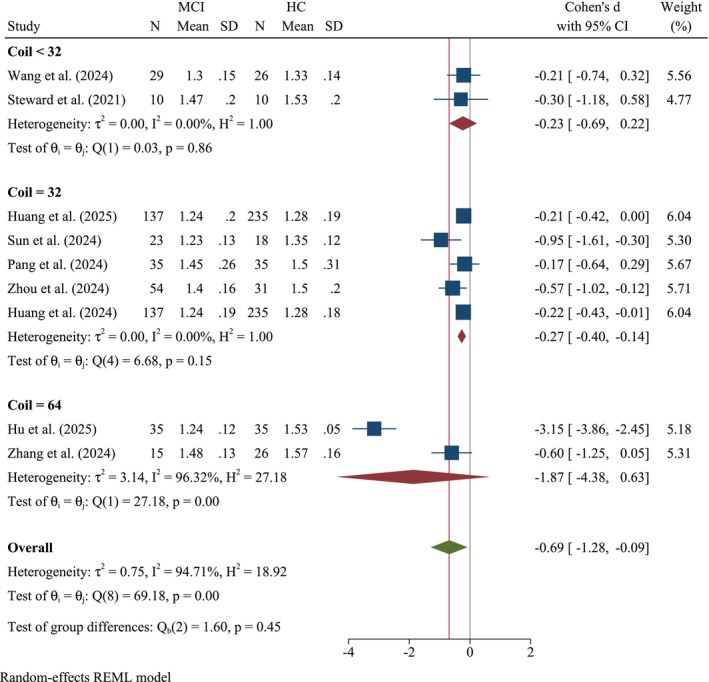
Forest plot of the meta‐analysis stratified by MRI coil channel.

Across the three coil‐size subgroups (< 32, = 32, and = 64) (Figure 6), Cohen's *d* indicated nonsignificant differences in the DTI‐ALPS index between MCI and HCs, except for studies with coil size = 32. In the coil < 32 subgroup, Cohen's *d* was −0.23 (95% CI: −0.69, 0.22, *k* = 2), with low heterogeneity observed (*τ* ^2^ = 0.00, *I* ^2^ = 0.00%; *Q*(1) = 0.03, *p* = 0.86). In the coil = 32 subgroup, the pooled effect was −0.27 (95% CI: −0.40, −0.14, *k* = 5), again with negligible heterogeneity (*τ* ^2^ = 0.00, *I* ^2^ = 0.00%; *Q*(4) = 6.68, *p* = 0.15). Conversely, the coil = 64 subgroup yielded a larger negative effect, Cohen's = −1.87 (95% CI: −4.38, 0.63, *k* = 2), but with substantial heterogeneity (*τ* ^2^ = 3.14, *I* ^2^ = 96.3%; *Q*(1) = 27.18, *p* < 0.001). A between‐group test confirmed that these subgroup differences did not reach statistical significance (*Q*_b_(2) = 1.60, *p* = 0.45), indicating that coil size did not systematically moderate the observed effects under a random‐effects REML model.

#### 3.2.5 Diffusion Directions Subgroup

**FIGURE 7:**
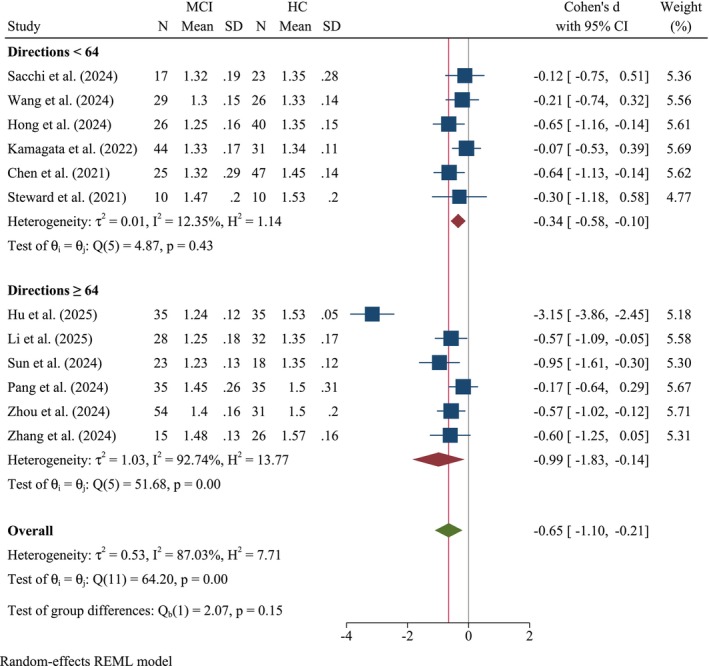
Forest plot of the meta‐analysis stratified by diffusion directions (cut‐off: 64).

A subgroup analysis stratified by the number of diffusion directions was performed (Figure 7). Studies utilizing diffusion directions < 64 yielded a pooled Cohen's *d* of −0.34 (95% CI: −0.58, −0.10, *k* = 6), indicative of a small‐to‐moderate reduction in the ALPS index. Low and nonsignificant heterogeneity was observed within this subgroup (*τ* ^2^ = 0.01, *I* ^2^ = 12.35%, *Q*(5) = 4.87, *p* = 0.43). Conversely, studies employing diffusion directions ≥ 64 demonstrated a larger effect size of −0.99 (95% CI: −1.83, −0.14, *k* = 6), reflecting a high decrease in the ALPS index, as well as low and nonsignificant heterogeneity (*τ* ^2^ = 1.03, *I* ^2^ = 92.74%, *Q*(5) = 51.68, *p* < 0.001). Despite the apparent difference in the SMDs, the test for subgroup differences (*Q*_b_(1) = 2.07, *p* = 0.15) indicated no statistically significant difference between the two groups.

#### 3.2.6 Male Ratio Subgroup

**FIGURE 8:**
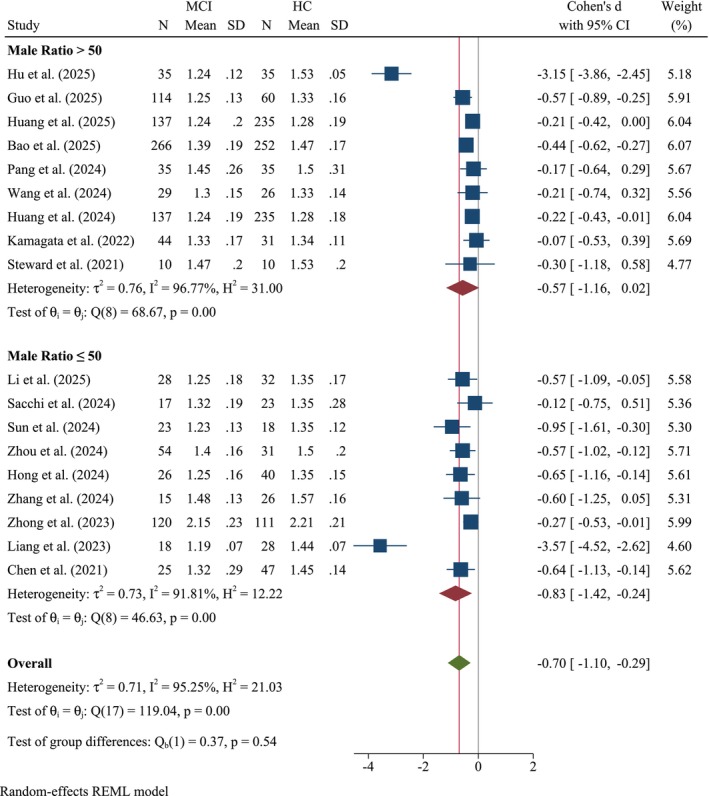
Forest plot of the meta‐analysis stratified by 50% male ratio.

Subgroup analysis stratified by a 50% male ratio revealed an interesting distinct glymphatic dysfunction pattern (Figure 8). For studies with a male ratio > 50%, Cohen's *d* was −0.57 (95% CI: −1.16, 0.02, *k* = 9), indicating a moderate but not statistically significant reduction in ALPS index values. Heterogeneity was high (*τ* ^2^ = 0.76, *I* ^2^ = 96.77%, *Q*(8) = 68.67, *p* < 0.001). In contrast, studies with a male ratio ≤ 50% demonstrated a stronger effect size (Cohen's *d* = −0.83, 95% CI: −1.42, −0.24, *k* = 9), reflecting a high decline in glymphatic activity with the same high heterogeneity (*τ* ^2^ = 0.73, *I* ^2^ = 91.81%, *Q*(8) = 46.63, *p* < 0.001). Notably, the test for subgroup differences was not statistically significant (*Q*_b_(1) = 0.37, *p* = 0.54).

#### 3.2.7 MMSE Subgroup

**FIGURE 9:**
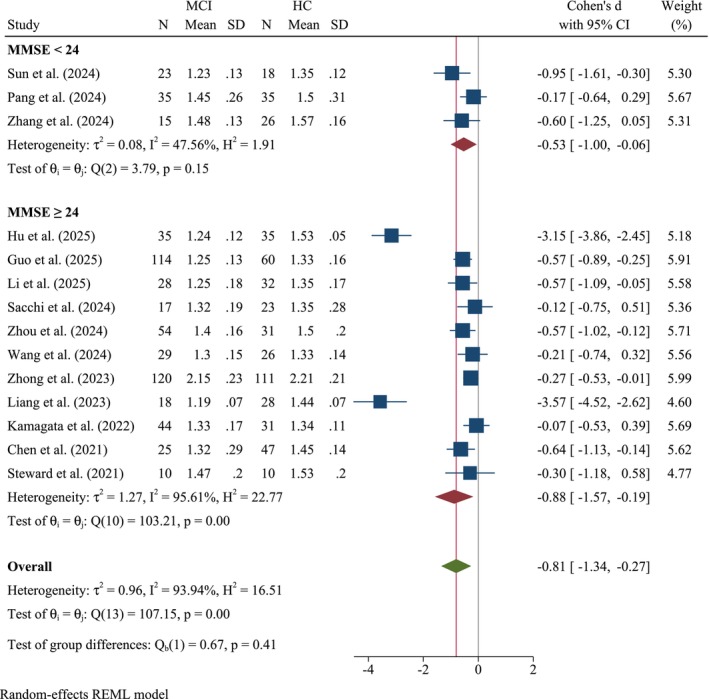
Forest plot of the meta‐analysis, stratified by MMSE scores.

Figure 9 presents a subgroup analysis of the association between MCI and DTI‐ALPS index stratified by MMSE scores. The studies were divided into two subgroups based on MMSE scores: MMSE scores < 24, indicating a greater degree of cognitive impairment, and MMSE scores ≥ 24, representing mild or no cognitive impairment. In the subgroup with MMSE < 24, Cohen's *d* was −0.53 (95% CI: −1.00, −0.06, *k* = 3). Moderate heterogeneity was observed in this subgroup but was not significant (*I* ^2^ = 47.56%, *Q*(2) = 3.79, *p* = 0.15). For the subgroup with MMSE ≥ 24, Cohen's *d* was −0.88 (95% CI: −1.57, −0.19, *k* = 11). This subgroup exhibited high heterogeneity (*τ* ^2^ = 1.27, *I* ^2^ = 95.61%, *Q*(10) = 103.21, *p* < 0.001). The test for subgroup differences (*Q*_b_(1) = 0.67, *p* = 0.41) indicated that the difference in effect sizes between the two MMSE subgroups was not statistically significant.

### 3.3 Publication Bias Results

**FIGURE 10:**
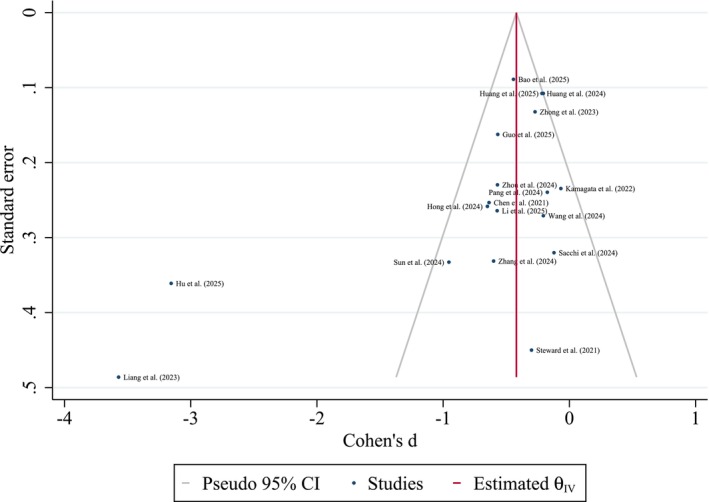
Funnel plot for publication bias assessment.

**FIGURE 11:**
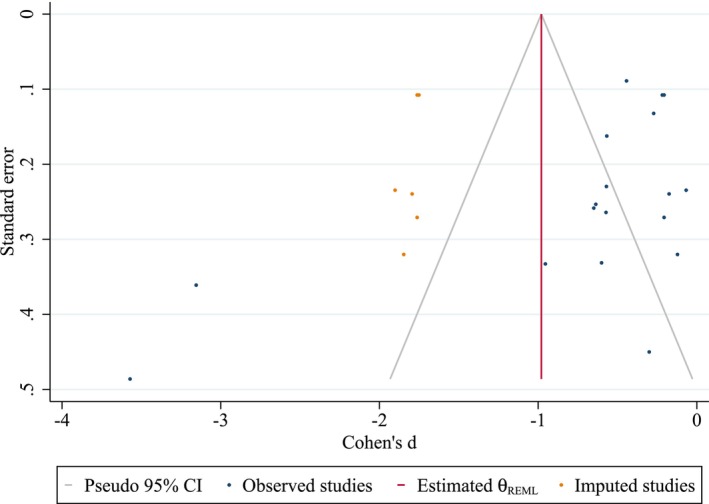
Trim‐and‐fill adjusted funnel plot (left‐side imputation).

Publication bias was assessed using Egger's and Begg's tests in addition to visual inspection of the funnel plot (Figure 10). Both Egger's test (*p* = 0.004) and Begg's test (*p* = 0.019) indicated statistically significant evidence of small‐study effects. The funnel plot visually supported this, displaying asymmetry, with a potential lack of studies showing smaller or nonsignificant effect sizes, particularly on the left side. Overall and right‐imputed trim‐and‐fill analysis did not add any potential missing studies and support our effect size robustness. However, the left‐imputed trim‐and‐fill analysis (Figure 11), imputing six potential missing studies on the left side of the funnel plot, resulted in a change of the pooled Cohen's *d* from −0.70 (observed) to −0.98 (observed + imputed) with a widened confidence interval, suggesting that the observed effect size might be an overestimation in the presence of publication bias favoring studies with larger negative effects.

### 3.4 Risk of Bias Results

The ROB assessment for the included studies, conducted using NOS adapted for cross‐sectional studies, demonstrated consistent methodological quality across studies (Table 2). All studies received scores > 7 (seven, *k* = 1; eight, *k* = 11; nine, *k* = 7), indicating low ROB. Most studies scored particularly well in the domains of ascertainment of exposure, control for confounding factors, and outcome assessment, consistently earning two stars in these categories. However, some studies, such as Liang et al. [62], did not account for nonrespondents or provide robust details regarding sample size, slightly lowering their total scores to seven and eight, respectively. Despite these variations, the overall ROB remained low, indicating reliable methodologies and results across studies included in the analysis.

**TABLE 2:** The Newcastle‐Ottawa Scale (NOS) adapted to the cross‐sectional studies for the risk of bias and quality assessment (ROB, risk of bias).

Author/year	Selection				Comparability	Outcome		Total score	Quality
	Representativeness of the sample	Sample size	Nonrespondents	Ascertainment of the exposure (risk factor)	Control for important or additional factors	Assessment of the outcome	Statistical test		
Hu et al. (2025) [50]	★	—	—	★★	★★	★★	★	8	Low ROB
Guo et al. (2025) [46]	★	—	—	★★	★★	★★	★	8	Low ROB
Huang et al. (2024) [51]	★	—	—	★★	★★	★★	★	8	Low ROB
Li et al. (2025) [52]	★	—	—	★★	★★	★★	★	8	Low ROB
Bao et al. (2025) [53]	★	—	—	★★	★★	★★	★	8	Low ROB
Sacchi et al. (2024) [54]	★	—	—	★★	★★	★★	★	8	Low ROB
Sun et al. (2024) [55]	★	—	★	★★	★★	★★	★	9	Low ROB
Pang et al. (2024) [56]	★	—	—	★★	★★	★★	★	8	Low ROB
Zhou et al. (2024) [57]	★	—	★	★★	★★	★★	★	9	Low ROB
Wang et al. (2024) [58]	★	—	—	★★	★★	★★	★	8	Low ROB
Huang et al. (2024) [51]	★	—	★	★★	★★	★★	★	9	Low ROB
Hong et al. (2024) [59]	★	—	★	★★	★★	★★	★	9	Low ROB
Zhang et al. (2024) [60]	★	—	—	★★	★★	★★	★	8	Low ROB
Zhong et al. (2023) [61]	★	—	★	★★	★★	★★	★	9	Low ROB
Liang et al. (2023) [62]	★	—	—	★★	★	★★	★	7	Low ROB
Kamagata et al. (2022) [63]	★	—	—	★★	★★	★★	★	8	Low ROB
Chen et al. (2021) [64]	★	—	★	★★	★★	★★	★	9	Low ROB
Steward et al. (2021) [65]	★	—	—	★★	★★	★★	★	8	Low ROB

## 4 Discussion

Our review provides substantial evidence to support the hypothesis that glymphatic dysfunction is present in individuals with MCI. As noted by Taoka et al. [36], a higher ALPS index exclusively reflects the predominant Brownian motion of water molecules in the radial direction at the lateral ventricular body level and nothing beyond that. A lower DTI‐ALPS index suggests impaired fluid movement along these spaces, indicating reduced glymphatic system function [38]. Our main findings align with the growing body of evidence suggesting a crucial role for decreased DTI‐ALPS, reflecting PVS glymphatic dysfunction, in the pathogenesis of neurodegeneration and as a predictor of cognitive decline [67, 68]. Our findings of reduced DTI‐ALPS in patients with MCI support the idea that glymphatic dysfunction is an early event in the progression of neurodegenerative diseases, potentially preceding or contributing to the development of dementia [51, 54, 56, 62, 69, 70, 71]. This is further supported by studies that found associations between lower ALPS index and the presence of the functional waste clearance pathway dysfunction, responsible for flushing solutes such as amyloid β and tau, metabolic, and other cellular waste products from the brain [51, 54, 70, 72, 73]. Furthermore, these findings are consistent with prior studies and reviews that have also reported decreased DTI‐ALPS indices in patients across the AD continuum, including MCI, as well as in other neurodegenerative conditions such as Parkinson's disease (PD) and PD patients with MCI and dementia compared to HCs, which is consistent with emerging studies [32, 70, 74, 75, 76, 77]. Furthermore, the novelty of the DTI‐ALPS method, first introduced in 2017 [38], explains the recent concentration of studies from 2021 onwards, as it took time for the technique to be adopted and applied to clinical cohorts.

Demographic, technical, and cognitive variabilities emerged as critical moderating factors. Subgroup analyses revealed intricate patterns in glymphatic dysfunction, as assessed via DTI‐ALPS. Older MCI patients (> 68 years) demonstrated a more substantial reduction in the ALPS index (−0.83 vs. −0.50 in ≤ 68 years), consistent with age‐related glymphatic decline, although the subgroup differences did not reach statistical significance. The selection of 68 years as a cut‐off was based on the median age of the pooled patient sample, a common statistical approach for creating balanced subgroups. However, this data‐driven split may have resulted in a relatively small age difference between the two groups, potentially contributing to the lack of statistical power to detect a significant subgroup effect.

Technical parameters significantly influenced the findings; studies employing ≥ 64 diffusion directions reported larger effect sizes, and investigations using 32‐channel coils revealed notable group differences. This tends to produce more robust and consistent findings, likely attributable to improved signal‐to‐noise ratios and spatial resolution [78, 79]. However, this highlights the importance of optimizing imaging protocols to enhance sensitivity to glymphatic alterations and underscores the need for standardized methodologies, such as coil and protocol optimization [36], including echo time selection, to improve cross‐study comparability [80]. Interestingly, subgroup analysis based on MMSE scores (cut‐off: 24) indicated a larger effect size in MCI patients with MMSE scores ≥ 24 (−0.88) than in those with MMSE scores < 24 (−0.53). This result appears counterintuitive [63, 81, 82], as more severe cognitive impairment (lower MMSE scores) is typically expected to correlate with greater glymphatic dysfunction. This finding is likely explained by the high overall mean MMSE score (25.42) of the included MCI patient population. The use of a cut‐off at 24 may have resulted in two subgroups with only a small true difference in cognitive function, thus limiting the statistical power to detect a significant difference in glymphatic impairment between them. Nonetheless, the difference between these subgroups was not significant (*p* = 0.41). The pooled studies reported a mean MMSE score of 25.42 for MCI patients, suggesting MCI. It is plausible that in the very early stages of cognitive decline or within specific MCI subtypes, the DTI‐ALPS index demonstrates greater deviation, or that additional confounding factors influence this relationship. For example, Kamagata et al. [63] identified associations between MRI‐derived glymphatic indices, amyloid deposition, and cognitive performance in MCI and AD populations. Sex‐based stratification revealed a trend toward larger effects in studies with ≤ 50% male participants, although the underlying biological mechanisms driving this observation remain unclear. This observation aligns with epidemiological evidence indicating a higher risk of MCI in females [83, 84, 85]. However, these findings should be interpreted with caution, particularly when evaluating sex differences (e.g., variations in head size) in glymphatic function [86]. It is important to note that this study‐level stratification by male ratio was an exploratory analysis, constrained by the lack of individual patient data. This method serves as an indirect proxy for sex differences and may not have created subgroups with sufficiently distinct sex compositions, which could contribute to the nonsignificant subgroup finding. Future meta‐analyses with access to individual participant data are needed to more definitively investigate sex‐specific differences in glymphatic function.

Complementing our findings, a recent Bayesian meta‐analysis confirmed the consistency of DTI‐ALPS in distinguishing patients with PD and AD from HCs, reporting strong evidence of its utility [86]. Longitudinal data further link ALPS to disease progression; a higher baseline ALPS predicts slower cognitive decline and delayed conversion from MCI to AD [53, 67]. Thus, aggregated findings indicate that glymphatic impairment emerges early in the pathogenesis of neurodegeneration and worsens with increasing disease severity. Another recent study found that both patients with MCI and AD had significantly lower ALPS indices than HCs, indicating reduced glymphatic activity [63]. In that cohort, a lower ALPS index correlated with AD biomarkers, which were associated with decreased CSF Aβ42 (suggesting greater brain amyloid) and reduced FDG‐PET uptake, as well as poorer cognition. A PET/MRI study [87] of early AD reported that the DTI‐ALPS index was inversely correlated with cortical amyloid (PiB‐PET centiloid) and positively correlated with MMSE. In fact, ALPS showed a stronger association with amyloid load than other MRI markers. Conversely, in a study of cognitively normal elderly individuals (with or without subjective memory complaints), ALPS did not differ by complaint status or overall amyloid positivity, although lower ALPS correlated with older age and higher amyloid levels in specific regions [88]. Importantly, a large longitudinal ADNI analysis found that a higher baseline ALPS index predicted a markedly lower risk of cognitive decline: individuals with a higher ALPS had a 40%–50% lower risk of developing MCI or AD over ~3–4 years [53], translating into an average 3–4‐year delay in disease onset. Finally, in patients with established AD, comorbid sleep disorders were linked to even lower ALPS indices and worse cognition, suggesting that sleep‐related glymphatic failure can exacerbate clinical decline [89]. Some inconsistencies in our findings suggest a need for further investigation to better understand the status of cognition and glymphatic system function.

A central factor in fluid dynamic impairment of the perivascular network is astrocytic AQP4 channel dysfunction [90]. Astrocyte aquaporin‐4 (AQP4) water channels on astrocyte endfeet normally facilitate periarterial CSF influx and interstitial solute clearance [2, 86]. In aging, MCI, and AD, AQP4 becomes depolarized or mislocalized (loss of polarized astrocytic distribution), which significantly slows glymphatic flow [90]. Experimental evidence has shown that AQP4 knockout in AD mouse models dramatically worsens amyloid accumulation and cognitive deficits [91, 92]. Depolarized or reduced AQP4 expression leads to impaired clearance of both Aβ and tau [92]. A primary study [2] used in vivo two‐photon imaging in mice to demonstrate the perivascular (paravascular) route of CSF influx and solute efflux. They found that injected fluorescent Aβ was rapidly transported along perivascular channels and that AQP4 knockout markedly slowed Aβ clearance. These findings suggest that glymphatic bulk flow normally removes Aβ from brain parenchyma. Conversely, insoluble Aβ can disrupt AQP4 polarization, creating a vicious cycle [92].

Based on recent editorials [93, 94] and our meta‐analysis, several limitations and potential future work of the current study must be acknowledged. A primary limitation of current glymphatic imaging research is the reliance on an indirect proxy that measures water diffusion along PVSs, which may not exclusively capture glymphatic clearance could also reflect broader microstructural alterations, such as white matter degeneration or vascular pathology. The dependence of this method on diffusion anisotropy introduces potential confounding factors, as changes in the tissue microstructure or the integrity of adjacent structures can inflate or obscure the true glymphatic signal. Additionally, many studies have employed cross‐sectional designs, precluding causal inferences regarding whether glymphatic dysfunction drives neurodegeneration or is a secondary consequence of other pathologies. Variability in imaging protocols, including differences in diffusion directions, *b*‐values, coil configurations, imaging planes, and echo times, further complicates comparisons across studies and may introduce measurement bias. Demographic heterogeneity, such as differences in age and sex, remains unaddressed, limiting our understanding of how hormonal influences, genetic risk factors, and lifestyle variables modulate glymphatic function. Anatomical challenges also persist, as current MRI techniques struggle to clearly distinguish lymphatic structures from adjacent venous channels or CSF fluid spaces, thereby complicating the interpretation of glymphatic–lymphatic coupling.

Future research should focus on longitudinal studies monitoring glymphatic dynamics over time to elucidate the temporal relationship between glymphatic dysfunction and neurodegenerative processes, including proteinopathy accumulation (e.g., amyloid‐β and tau) [54]. Standardization and harmonization of diffusion MRI protocols are critical for improving the reliability and comparability of findings, and the development of automated or semiautomated delineation methods could help reduce observer bias. Integration with other methods (e.g., PET amyloid imaging or CSF biomarkers) [51, 87], genome‐wide association [72, 95], and computational approaches [77] may enhance diagnostic accuracy and mechanistic insights. Additionally, integrating multimodal and multiparametric imaging approaches, including blood‐oxygen‐level‐dependent CSF coupling [96], quantitative susceptibility mapping [97], and DKI‐ALPS [98] for non‐Gaussian analysis, could provide a more comprehensive understanding of how glymphatic function interacts with structural and functional brain networks. Exploring pharmacological and lifestyle interventions to enhance glymphatic clearance and developing new diffusion methods to address issues such as crossing fibers (e.g., a three‐axis DTI‐ALPS technique) will be crucial for advancing the field and ultimately tailoring therapeutic strategies to mitigate cognitive decline.

The current analysis focused specifically on the comparison between MCI and healthy controls. While several included studies also featured cohorts with Alzheimer's disease or Parkinson's disease dementia, a direct meta‐analytic comparison across different disease stages was beyond our scope. Future meta‐analyses should aim to compare the ALPS index across the continuum of healthy aging, MCI, and dementia to investigate a potential gradient of glymphatic dysfunction, which would provide a more comprehensive understanding of its role in disease progression.

## 5 Conclusions

These findings underscore the value of the ALPS index as a noninvasive biomarker for detecting early glymphatic dysfunction associated with cognitive decline as aging progresses. This study strengthens evidence that the DTI‐ALPS index is a reliable and early indicator of glymphatic dysfunction in individuals with MCI. Our meta‐analysis suggests a potential interplay of aging, technical variability, and cognitive heterogeneity in shaping glymphatic measurements, emphasizing the need for standardized protocols and a deeper investigation into confounders, such as vascular risk factors, MCI subtypes, and sex‐specific fluid dynamic differences.

## Author Contributions

S.G.: conceptualization, methodology/study design, data curation, writing – original draft preparation, visualization, investigation, supervision, software, formal analysis, validation, writing, reviewing, and editing. S.M.: conceptualization, methodology/study design, writing – original draft preparation, visualization, investigation, supervision, software, formal analysis, validation, writing, reviewing, and editing. A.F.J. and A.M.A.: data curation, writing – original draft preparation, visualization, investigation, and validation. All authors have read and approved the final version of the manuscript.

## Funding

The authors have nothing to report.

## Ethics Statement

The authors have nothing to report.

## Conflicts of Interest

The authors declare no conflicts of interest.

## Supporting information


**Figure S1:** Sensitivity analysis of the meta‐analysis results.
**Table S1:** The search strategies used for database searches (05/10/2025).

## Data Availability

The data that support the findings of this study are available from the corresponding author upon reasonable request.
