# Supplementary material for: Glymphatic Pathway Dysfunction in Mild Cognitive Impairment: A Systematic Review and Meta‐Analysis Using Diffusion Tensor Imaging Along the Perivascular Space
Source: CNS Neurosci Ther. 2025 Dec 19;31(12):e70695. doi: 10.1002/cns.70695 (PMC12715781; doi:10.1002/cns.70695)
Supplement: Supplementary file 1 — Figure S1: Sensitivity analysis of the meta‐analysis results. Table S1: The search strategies used for database searches (05/10/2025). [file CNS-31-e70695-s001.docx]

| **Table S1.** The search strategies used for database searches (05/10/2025) | | |
| --- | --- | --- |
|  | **Database** | **N** |
| PubMed | ("Glymphatic*"[Title/Abstract] OR "Glymphatic system"[Title/Abstract]) AND ("DTI"[Title/Abstract] OR "DTI-ALPS"[Title/Abstract] OR "Diffusion*"[Title/Abstract]) AND ("Mild Cognitive Impairment"[Title/Abstract] OR "MCI"[Title/Abstract]) | 21 |
| Scopus | TITLE-ABS-KEY(("glymphatic" OR "glymphatic system")  AND  ("DTI" OR "DTI-ALPS" OR "diffusion")  AND  ("mild cognitive impairment" OR "MCI")) | 36 |
| Web of Science (WOS) | TS=("glymphatic" OR "glymphatic system")  AND  TS=("DTI" OR "DTI-ALPS" OR "diffusion")  AND  TS=("mild cognitive impairment" OR "MCI") | 27 |
| Embase | ('glymphatic' OR 'glymphatic system')  AND  ('DTI' OR 'DTI-ALPS' OR 'diffusion')  AND  ('mild cognitive impairment' OR 'MCI') | 44 |


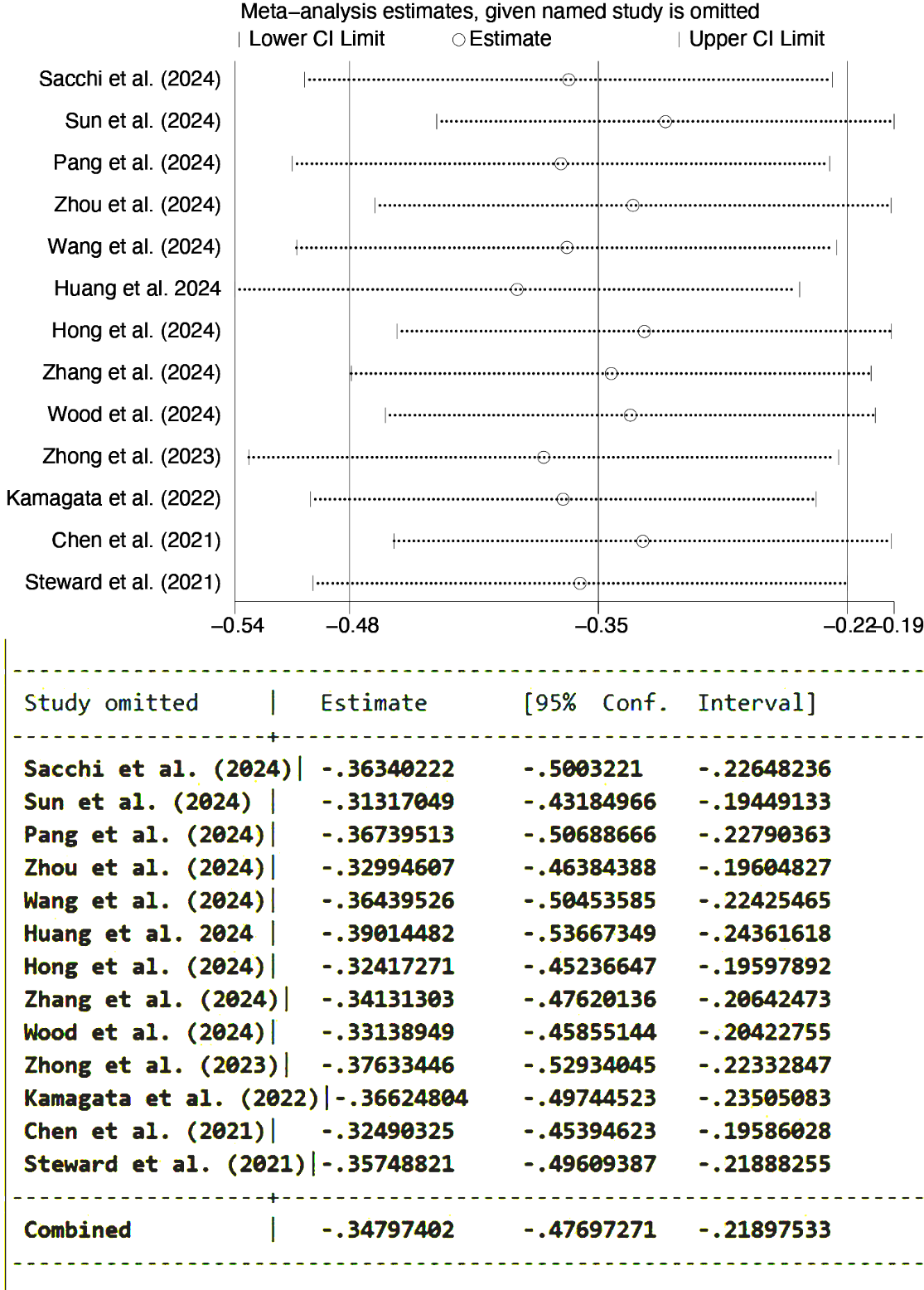


**Figure S1.** Sensitivity Analysis of the Meta-Analysis Results
